# Supplementary material for: Reconciling Mining with the Conservation of Cave Biodiversity: A Quantitative Baseline to Help Establish Conservation Priorities
Source: PLoS One. 2016 Dec 20;11(12):e0168348. doi: 10.1371/journal.pone.0168348 (PMC5173368; doi:10.1371/journal.pone.0168348)
Supplement: S1 Dataset — (ZIP) [file pone.0168348.s002.zip › Taxa/Serra Norte/SN_2013/N4E/Lista N4E-82.pdf]

CAVIDADE N4E-0082

Classe	Ordem	Fam/Outros	Gên/Outros	Espécie	S-U	U-S	Zon.
Arachnida	Amblypygi	Charinidae	*Charinus*	sp.1	1		P/A
Arachnida	Araneae	Araneidae	*Alpaida*	*antonio*		X	E
Arachnida	Araneae	Caponiidae		sp.1	X		P/A
Arachnida	Araneae	Pholcidae	*Leptopholcus*	sp.1	X	X	E
Arachnida	Araneae	Pholcidae	*Mesabolivar*	sp.1	X		E/P
Arachnida	Araneae	Theridiosomatidae	*Plato*	sp.1	X		E/P/A
Arachnida	Araneae	Uloboridae		jovem	X		E
Arachnida	Opiliones	Escadabiidae		sp.2	X		P/A
Arachnida	Opiliones	Stygnidae	*Protimesius*	*gracilis*		1	E
Diplopoda	Polydesmida	Chelodesmidae		jovem		1	E
Diplopoda	Polydesmida	Fuhrmannodesmidae		sp.1	X		P/A
Diplopoda	Polydesmida	Pyrgodesmidae		jovem	X		P/A
Entognatha	Collembola	Entomobryidae		sp.1	X		E
Entognatha	Collembola	Paronellidae		sp.4	X		P/A
Insecta	Blattodea	Blattidae		sp.3	1		
Insecta	Coleoptera	Dytiscidae		sp.18	X		E/P
Insecta	Diptera	Cecidomyiidae	Cecidomyiinae	sp.	X		E
Insecta	Diptera	Chaoboridae		sp.		X	E
Insecta	Diptera	Chironomidae		sp.	X		E
Insecta	Diptera	Culicidae	*Culex*	sp.	X		E
Insecta	Diptera	Mycetophilidae		sp.	X		E
Insecta	Diptera	Psychodidae	Phlebotominae	sp.	X		P/A
Insecta	Diptera	Tipulidae	Tipulinae	sp.		X	E
Insecta	Heteroptera	Gerridae	*Tachygerris*	jovem	X	X	E
Insecta	Heteroptera	Veliidae	*Microvelia*	sp.	X	X	E
Insecta	Hymenoptera	Formicidae	*Paratrechina*	sp.1	X		P/A
Insecta	Hymenoptera	Formicidae	*Camponotus*	sp.1	X		P/A
Insecta	Hymenoptera	Formicidae	*Dolichoderus*	aff. *bispinosus*	X		E
Insecta	Hymenoptera	Formicidae	*Pachycondyla*	sp.2		X	E
Insecta	Hymenoptera	Formicidae	*Paratrechina*	sp.1		X	E
Insecta	Hymenoptera	Formicidae	*Paratrechina*	sp.2	X		P/A
Insecta	Hymenoptera	Formicidae	*Pheidole*	sp.1	X		E
Insecta	Isoptera	Termitidae	*Nasutitermes*	sp.	X		E
Insecta	Orthoptera	Phalangopsidae	*Paraclodes*	jovem		1	E
Insecta	Plecoptera			jovem	4		P
Gastropoda	Pulmonata	Bulimulidae	*Naesiotus*	sp.	X		P/A
Malacostraca	Isopoda	Scleropactidae	*Circoniscus*	*buckupi*	X		P/A
Amphibia	Anura			sp.	35	1	E
